# Choosing Fitness-Enhancing Innovations Can Be Detrimental under Fluctuating Environments

**DOI:** 10.1371/journal.pone.0026770

**Published:** 2011-11-17

**Authors:** Julian Z. Xue, Andre Costopoulos, Frederic Guichard

**Affiliations:** 1 Department of Biology, McGill University, Montreal, Canada; 2 Department of Anthropology, McGill University, Montreal, Canada; University of Sheffield, United Kingdom

## Abstract

The ability to predict the consequences of one's behavior in a particular environment is a mechanism for adaptation. In the absence of any cost to this activity, we might expect agents to choose behaviors that maximize their fitness, an example of directed innovation. This is in contrast to blind mutation, where the probability of becoming a new genotype is independent of the fitness of the new genotypes. Here, we show that under environments punctuated by rapid reversals, a system with both genetic and cultural inheritance should not always maximize fitness through directed innovation. This is because populations highly accurate at selecting the fittest innovations tend to over-fit the environment during its stable phase, to the point that a rapid environmental reversal can cause extinction. A less accurate population, on the other hand, can track long term trends in environmental change, keeping closer to the time-average of the environment. We use both analytical and agent-based models to explore when this mechanism is expected to occur.

## Introduction

One of the distinguishing features of biological evolution is the production of adaptation through natural selection on blind variation [1]. A vast amount of theory and empirical work has been dedicated to this topic, stemming from the earliest works of Fisher [2] and other classical population geneticists (e.g. [3], [4]). However, this mechanism is not the only method by which adaptation can be produced.

Population genetics is fairly sparse on the subject of directed adaptation, or the overproduction of new phenotypes that adapt the organism well to the current environment. This is, of course, because genetic mutation is blind. However, the behavioral innovations of some animals are not necessarily blind. Some animals can predict the consequences of their actions within their environment, which increases their probability of choosing a behavior or innovation among a large pool of possibilities that fit them well to the current environment. These behaviors can then be transmitted via cultural inheritance to the next generation. Thus they can adapt behaviorally in a directed way.

Intuitively, we can believe that such directedness promotes the maintanence of high fitness. In fact, the scholarly work on the capacity for predicting the consequences of our actions is littered with optimistic statements, going all the way back to Malthus, who spoke of:

“…that distinctive superiority in [human] reasoning faculties, which enables [them] to calculate distant consequences” [5].

The intution that being able to predict the consequences of one's actions is good for fitness remain today in the works of most authors on this topic (e.g. [6], [7] and comments in [7]). For example, D'Argembeau in a comment to [7] says:

“S&C [Suddendorf and Corballis] argues that the primary fitness function of mental time travel is to enhance biological fitness in the future: Mentally simulating various versions of the future, and their respective consequences, enables one to act flexibly in the present to increase one's future survival chances. We completely agree…”

The reasoning seems to be that agents with the ability to predict the consequences of their action in the environment can choose the best possible action out of the pool of all possible actions, and thereby increase its adaptedness, and therefore its fitness. However, these predictions ignore the effects of environmental fluctuations, including sudden changes in fitness optima, which often have counterintuitive consequences for evolution [8], [9]. One would like a more rigorous argument of when the directedness of innovations is advantageous, as well as quantitative predictions of just how advantageous [10]. After all, it is reasonable to assume that the capacity for directed innovation increases with brain size, which is expensive in terms of energy [11], obstetric difficulties [12], and a long childhood [13]. A clearer theoretical understanding would allow us to predict trade-offs involving directed innovation, as well as the situations in which directed innovation can be more, or less, important.

Here, we derive a scenario where maximizing one's accuracy in choosing the fittest innovation in the current environment can be detrimental. The systems we study are those with dual inheritance [14], where the parent is able to pass on both genes as well as learned behavior. Our approach is based on the intuition that agents that are too good at choosing fitness-enhancing innovations can overfit environmental change. This possibility has already occurred to Darwin, who wrote that

“If animals became adapted to every minute change, they would not be fitted to the slow great changes really in progress.” [15].

Thus, agents who are capable of assessing the consequences of their own actions in the immediate environment, but who are incapable of forecasting the changes of the environment, might be thrown off by environmental noise rather than adapting themselves to long term trends or time-averages. We more specifically show how the occurrence of sudden changes in the environment can limit the degree of directed innovation. We first provide an intuitive description of this scenario. We then present a formal analytical treatment, and finally we report results from an agent-based simulation applied to a historical example to which our scenario might apply.

## Models and Results

### Toy model

**Figure 1:**
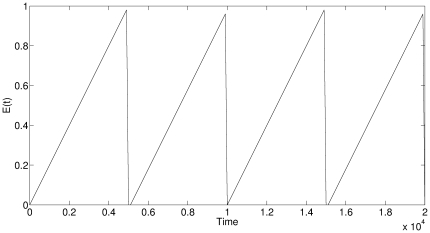
An artificial environment used by the agent based model. *E*(*t*) represents the environmental value at time *t*.

Let us begin with an oversimplified toy model in order to formalize this intuition. Consider an environment, characterized by a single parameter *E* that varies around a certain mean. In fact, let us consider a very particular type of environment, where *E* slowly increases over a long period of time, then quickly reverses and crashes (see Figure 1).

Let's say there are two agents exposed to *E* and there is a pool of actions from which each of the agents can choose. Different actions alter their fitness in response to *E*. However, the pair of agents differ in their ability to assess the consequences of any particular action in the environment. We call the one who can assess better the “accurate” agent and the one who assess less well the “inaccurate” agent. Note that this is not yet a model of evolution, rather of competition between two agents who do not die. The competition is based on how well the agents learn from the environment.

We model the capacity for directed innovation as the average reduction of the distance between individual fitness and the fitness optimum following each innovation. This is intutively reasonable because the agents who can assess better can change its behavior to be more in tune with changes in the environment, and hence produce better adaptations. The inaccurate agent, on the other hand, cannot adapt its behavior to environmental change so well.

Consider the case where the inaccurate agent is so poor that, on average, its innovations cannot even track the enviromental changes during the phase of slow increase in , and remain near the long term environmental mean despite the slow environmental change. Thus, for the period of slow environmental change, the accurate agent will do better. However, when the environment rapidly reverses, the accurate agent might in fact do worse. It might do worse because it fits the previous change so well that the rapid reversal meant the environment moved further away from its optimum than the optimum of the inaccurate agent, which had stayed near the environmental mean.

We can reason out the conditions allowing inaccurate types to maintain a higher long-term fitness than accurate types. First of all, if the accurate agent is able to perfectly adapt to the phase of rapid enviromental change, then it will always have a higher fitness than the inaccurate agent. The perfect strategy for agents is to perfectly track the environment. However, this strategy is impossible if environment changes so rapidly in its rapid phase as to escape the capacity of both types of agents to adapt.

Secondly, the environment must possess a strong central tendency and be varying about some longer-term mean, or trend, since if its rapid phase is in the *same* direction as the slow phase, then the accurate agent will be superior. Finally, the fitness function must be non-linear in the sense that the cost to the accurate agent after the rapid environmental reversal must outweigh the fitness advantage it has accumulated over the period of slow environmental change.

The first assumption, that perfect adaptation is impossible, seems to be a reasonable one in view of how the environment changes at all scales of time. It is impossible for organisms to adjust themselves to every minute change that occurs with great rapidity. The second one also seems reaonable, since most environmental conditions do vary about a certain mean, although whether they change slowly in one direction and then quickly in another depends on the scenario at hand, and we will give an example in the final section.

The third assumption is more subtle. Superficially, it already seems quite reasonable, since most fitness curves are convex near the optimum [16]–[19]. The usual theoretical curve used here is a gaussian curve, so both theoretically as well as empirically the fitness drops more precipitously as the agent's phenotype moves away from the enviromental optimum. Thus, a short moment of severe maladaptation can strongly outweight long periods of relative better adaptation. On the other hand, a more subtle theoretical point is that the curvature of the fitness curve is not necessary, although a strong curvature makes the less accurate type much more likely to be more fit. This is due to the well-known principle that natural selection maximizes the geometric mean fitness over time, not the arithmetic mean [20]–[23]. Thus, while maximizing fitness, natural selection also minimizes variance in fitness over time, and since the worst fitness of the accurate agent is worse than the worst fitness of the inaccurate agent, the accurate agent accrues more variance in its fitness, lowering its geometric mean fitness over time.

We next construct an analytical model to provide a formal theory of maladaptive directed innovation under sudden environmental changes.

### Analytical model

The above section describes a toy model in which maximizing the fitness of each decision may not be an optimal strategy when the environment changes slowly in one direction for long periods and then rapidly reverses. This can be illustrated with a numerical example. Consider a simple cyclical environment with cycle 4 that looks like the following: 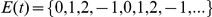, where 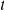 indicates the timestep.

Consider two agents, 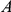 and 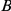, where 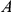 is more accurate than 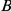. Due to greater accuracy, 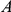 can adapt to the environment more quickly than 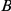 by choosing better actions. Let us say, for example, 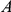 can change its optimal phenotype by a maximum of 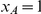 per time step in the direction that will take its phenotype closer to the environmental optimum. 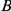, on the other hand, is incapable of choosing so well and 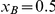. Let both agents be perfectly adapted to the enviroment initially, and let 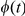 be the phenotype of the agent at time 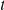. The phenotype is measured as the environment to which the organism is best adapted. We thus set 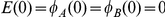.

We give the first few entries of the sequence of 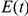, 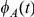, and 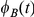 for clarity:
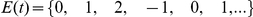


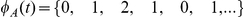


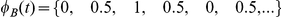



We introduce the fitness curve
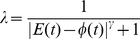
(1)where 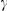 is a tunable parameter. This fitness curve looks a great deal like a Gaussian curve, but is easier to compute with (Figure S1, see Information S1). 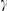 controls the rate at which the curve falls off as 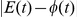 increases; a greater 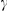 means a sharper threshold of 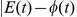 at which fitness falls off precipitously.

It turns out that 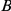 is more fit than 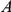 for 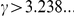. In fact, the existence of some critical 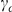 is true in general; for similar environments and fitness curves, then an agent 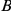 with less foresight than agent 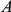 will be more fit than agent 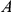 if 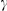 is large than some 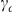 (see Information S1). Moreover, we can show that for any such environment (such as the one in Figure S2), agents behave cyclically and there is an intermediate value of 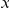 which is locally optimal for any 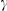. (See Figure S3, Information S1).

### Agent-based model

Our model of directed innovation differs from models of blind mutation. In blind models, mutations themselves do not take the agent closer to the environmental mean. Any movement towards the environmental mean is due to the differential effects of natural selection. In the above analytical model, however, the directed movement is purely due to the capacity of the agent to choose, on average, an innovation that takes it closer to the environment. The previous model is not a model of evolution. We did not include variance in innovations and there was no reproduction or selection.

The analytical model shows that agents with less directedness can be more fit than agents with more directedness. In order to make the model evolutionary, we extend the above analytical model with a dual inheritance agent-based model, where the agent's behavior begins as being learned (culturally inherited) from its parent, then changes according to its genetically determined capacity to learn from the environment, which is genetically inherited from the parent. This allows us to evaluate the likelihood of mutating off the local optimum, severity of conditions required on 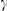 (if any), robustness to drift effects, limited population sizes, and the applicability of this theoretical scenario to the real world. We confirm that the intermediate accuracy under the conditions described by the analytical model is a robust prediction under these scenarios.

In the agent-based model, as in the analytical model, individual agents have a phenotypic value (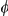) that represents their adaptation to an environment. The environment is uniform with one dimension of variability described by 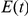. At each time step, behavioral innovations become available. Agents can adopt or refuse these innovations and as a result move their phenotype closer or farther away from the environmental optimum, increasing or decreasing their fitness. Agents have a second trait (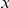) that represents their probability of knowing how behavioral innovations will affect them. Accurate agents (higher 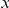) are better at determining whether any behavioral innovation will move their phenotypic value closer to the current environmental value. Inaccurate agents have lower 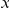. Agents adopt the behavioral innovation if and only if they can determine that it is beneficial. The probability of adoption is 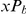, where 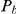 is the probability that a new behavior is beneficial. Agents reject the behavioral innovation if they are unable to determine whether it is beneficial, or if they determine it to be harmful. The assumption that new behaviors are always rejected if agents cannot foresee their consequences reduces the noise in simulation results. Otherwise inaccurate agents would have a great range of fitnesses (depending on what behaviors they adopt by chance), and these fitnesses would be independent of their foresight. This essentially turns the model into one of blind mutation, which we would like to avoid.

Consider 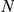 agents over the time period 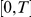, and the environment changes as 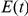. Each agent 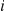 is defined by two parameters, 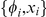, where 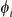 is the environment that 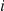 is adapted to, and 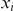 is the probability that agent 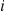 can assess the consequences of adopting a behavioral innovation, that is, whether the new behavior will move the agent closer or farther from the environmental optimum. At each timestep, there is a probability, 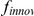, that a new behavior will be available for adoption. This behavior will increase or decrease 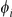 by 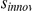 with equal probability (so 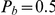) if adopted by any given agent. 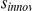 thus gives an upper bound to the maximum rate of adaptation to the current environment.

At each timestep, 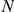 agents are chosen with replacement for update. During each update, agent 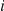 survives with the probability
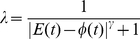
(2)


We used survival instead of reproductive fitness to increase simulation speed, but preserve the same qualitative behavior of the fitness curves [24]. The dynamics of the model are robust to implementation scheme; for example, a random agent can be chosen to replicate with this fitness, pushing out another random agent.

In our implementation, if agent 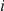 does not survive, a random agent 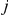 (not including 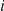) is chosen from the population to reproduce. Its progeny then replaces 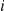. Thus, a new agent 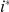, a progeny of agent 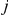, replaces the agent 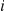 that has just died. The new agent 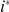 is very similar to 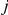 with small modifications. Specifically, 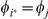, which is an act of cultural transmission (the passage of a learned behavior from parent to progeny), and 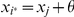, an act of genetic transmission with mutation, where 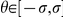, a uniform distribution. 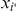 is a probability that is kept bounded between 0 and 1. We do not allow mutations in the culturally learned behavior because we wish for all movement of the phenotype towards the environmental mean to be a result of directed innovations by choice of the agents, rather than blind chance and selection.

If agent 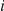 survives and a new behavior is available, it has 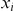 chance of accurately assessing whether the new behavior will take it closer to the environmental optimum. If it assesses successfully, and the new behavior moves the agent closer to the environment, then it adopts the behavior and modifies its 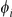. Else it does not adopt the new behavior. This is how we model directed innovations.

**Figure 2:**
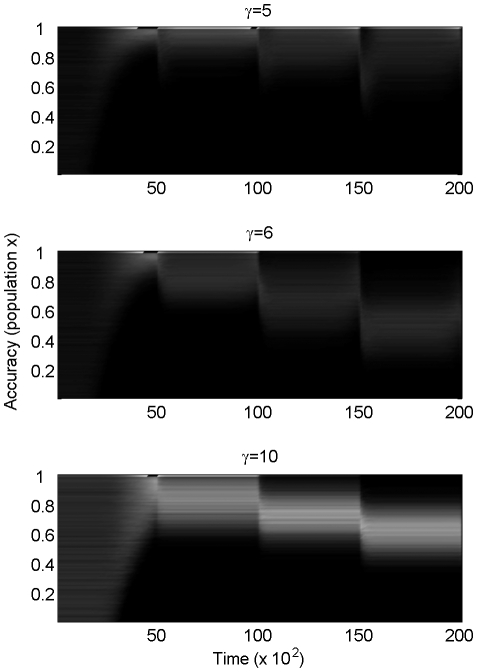
Intermediate adaptive rate (*x*) is optimal for agents in the environment of **Figure 1**. Brightness denotes density of agents. From top to bottom: 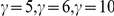. All other parameters are identical (see text for more details). Initial conditions: 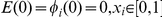. The results are independent of initial conditions. Note how the larger the gamma, the stronger the movement to intermediate, rather than maximal, accuracy.

We tested the model with a variety of environments. We report the results with an artificial environment (Figure 1) and one based on the Vostok ice core data [25], [26] (see next Section). In order to make the different environments comparable, we normalize them so the the minimum environmental value is 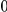 and the maximum environmental value is 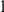. We examine a range of 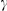
's and we note the minimum 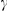 value for which the agents settle into an intermediate, rather than a maximal ability to foresee the consequences of new behaviors. For all simulations, 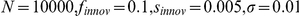. For the artificial environment presented in Figure 1, predicted 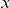 values are independent of initial conditions for all 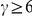. The results for 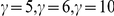 are presented in Figure 2. As expected from the analytical model, for large enough 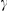 (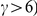
agents do not evolve to maximum possible foresight. Each sudden environmental reversal will favour anew the inaccurate agents.

This result depends on the assumption that even maximum foresight is not sufficient to perfectly track the environment during the sudden reversal. If perfect tracking was possible, the agents that could perfectly track would necessarily be the most fit. We argued in our Introduction that the inability to perfectly track the environment during sudden environmental changes is a reasonable assumption, and we will return to this assumption in the Discussion.

The main differences between the agent-based and the analytical models presented here are the dissociation between 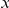 and 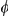, and limited population size introduced in the agent-based model. If the environment slowly rises in one direction for too long, or if 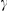 is too small, then all agents with low 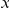 die before the onset of sudden environmental change, in which case high 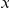 dominates, or the population goes extinct (which is prevented in this model). The minimum 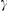 values needed to generate intermediate optimal 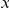 from the model means that we need a fitness curve that drops off quickly when the distance between the phenotype and the environmental optimum reaches a threshold. This way, inaccurate agents can survive until the catastrophic event (since they do not cross that threshold), but the catastrophic event is sufficient to kill off most of the accurate agents (since they do cross that threshold). Thus, although the analytical model predicts a locally optimal 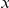 for all 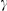, a minimum 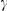 is required in the agent-based model to observe a local optimum.

### Agent-based modeling using the Vostok ice core data

**Figure 3:**
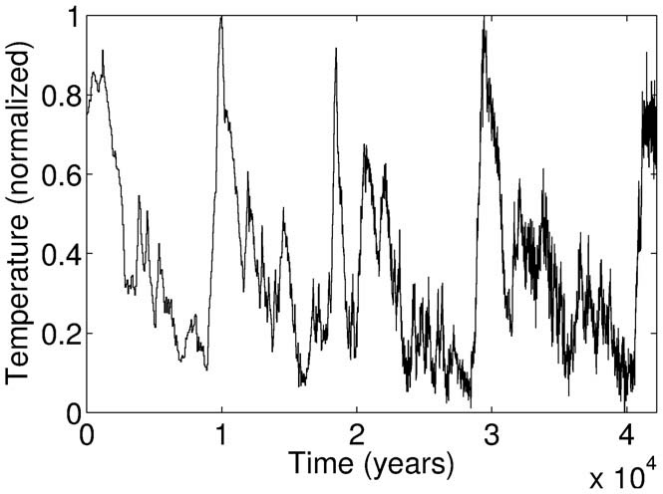
The Vostok ice core data. Long periods of cooling are punctuated by rapid deglaciation.

**Figure 4:**
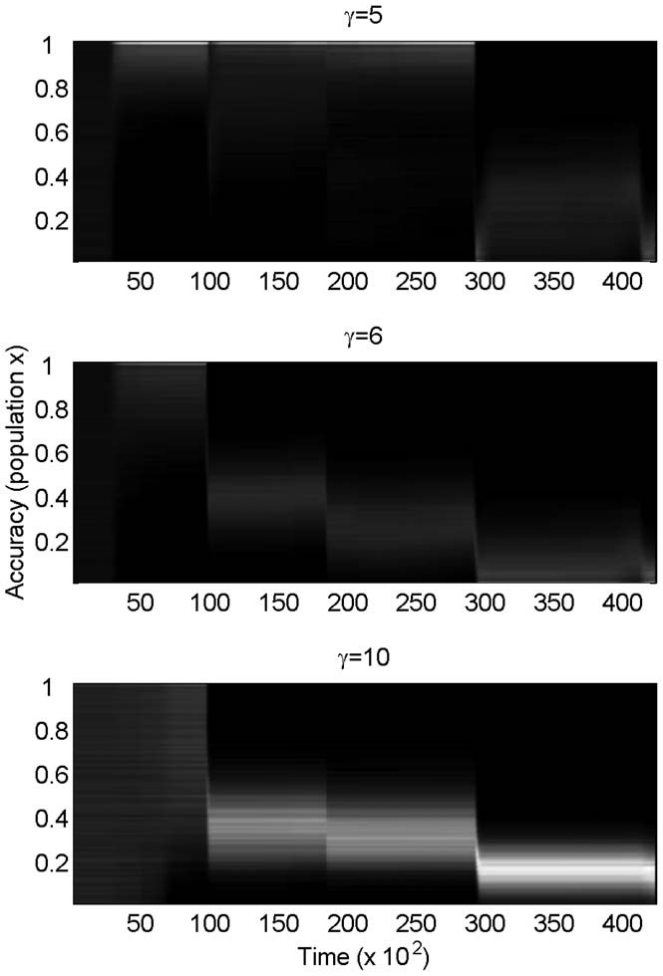
Agent discrimination ability using the Vostok ice core data. From top to bottom: 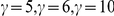. Notice how discriminatory ability rises during periods of slow change, but falls each time there is a rapid reversal.

The artificial environment we use in the previous section can be argued to be too artificial; the rise and the fall are both clean of noise and the sudden reversal might be too sudden and too quick to be realistic. To address this issue, we insert a more realistic timeseries in the form of the Vostok ice core data [25], [26]. This data indicates that the temperature environment over the last 400 000 years has been characterized by rapid deglaciation events, interspersed across periods of slower cooling and cold plateaus. We test whether this could result in an intermediate, locally optimal adaptive rate. We replace our artificial environment with the Vostok ice core data (Figure 3), where each timestep counts for a single year. All other details remain identical to the previous model. Even 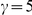 in this model favored agents of moderate or even low ability to foresee the consequences of adopting different behaviors (Figure 4). As expected, the effect increases with increasing 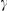.

The first significant period of change is a rapid glaciation 400kya that causes inaccurate agents to have a fitness advantage. Four rapid deglaciation events 350kya, 250kya, 150kya, and a last one in the past 20ky, all allow low inaccurate agents to persist for long periods. In other words, there is an advantage to reducing the overall ability of the agents to discriminate between beneficial and harmful innovations under these circumstances. We believe that a large range of environmental variables display this pattern of slow movement in one direction interspersed with catastrophic reversals, which potentiates the type of scenario described in this paper. Natural disasters, for example, might happen quickly, followed by long and slow periods of recovery.

## Discussion

In this paper, we show a somewhat counterintuitive point that agents which can best assess the consequences of their actions in the current environment can nevertheless be at a fitness disadvantage relative to less accurate competitors. We reason that the ability of agents to predict the consequences of their actions in a given environment acts like a greedy algorithm; it constantly tries to optimize for the moment. In the cases reported here, the greedy algorithm fails because the accurate agent over-optimizes for the immediate present, and so fails to track the long term average of the environment.

Several veins of earlier theoretical work shed light on these results. The first is the work on optimal rates of mutation (e.g. [27]–[30]). Another related line is natural selection and mutation rates in changing environments (e.g. [21], [31], [32]). The first body of work established that in the majority of cases, a low or zero mutation rate is preferable since the vast majority of mutations are deterimental. The fact that mutation rates are not zero is likely due to the physiological cost of increasing copying fidelity ([30]), especially in sexual beings where the hitchhiking of alleles that increases mutation rate to beneficial alleles is broken by recombination. The second line of work established the manner in which environments must change in order to maintain genetic variability (e.g. [32]). These results, however, are primarily focused on blind mutations. Directed innovations, where a larger proportion of change might be beneficial, require a different sort of analysis.

The closest work focuses on the evolution of individual learning (e.g. [8], [9], [14], [33]). The evolution of learning from the environment is strongly related to this work, since this type of learning (as opposed to social learning from other agents) can be seen as the act of choosing new behaviors with feedback from the environment. Most of this work, however, has focused on the evolution of the capacity for culture. A recent result that studies learning in variable environments, for example, showed that in a stable environment punctuated by sharp bouts of change, agents stop learning from the environment during the stable periods and instead opt to choose to learn from each other. When the environment does change, the agents can no longer learn from the environment and may go extinct [9]. Our work instead focuses purely on the dynamics of the evolution of learning from the environment and on the optimal rates of such learning. Previous work assumed that better capacity for learning, if costless in other fitness terms such as time and energy, would likely lead to improvement of agent fitness, although our results have been foreseen by [34], who realized that changes in environmental conditions can lead to a reduction of learning.

Our model applies to systems of dual inheritance, where the learning done by the parent generation is passed on to the progeny through cultural transmission, and the capacity to learn further from the environment is passed on through genetic transmission. The model also contains three key simplifying assumptions that allow for analytical tractability and increase the generality of our results. First is that agents cannot fully adapt to the environment using directed innovation when environmental change is at its quickest. This, we argue, is reasonable. The environment fluctuates on every timescale; even organisms with learning cannot adapt to every change. In the absence of prior adaptation, the maximum rate of environmental change during a volcanic eruption, for example, must be very quick indeed and no cognitive capacity is likely to cope with it in an optimal way. A recent review of the evolution literature [35] falls in very neatly with this assumption, that the environment constantly exerts considerable selection pressure on organisms, but with many changes in direction and with longer trends of change that proceed at a much slower pace.

The second assumption is that agents are incapable of forecasting the environment. Forecasting denotes agents' knowledge of the future environment. If there are agents with good environmental forecasting abilities who can know the environment many timesteps in advance, they can preadapt and nullify these theoretical predictions. Future explorations will allow us to understand the sensitivity of this model to forecasting. However, we expect our results to be robust to some amounts of forecasting, which would be consistent with overall difficulty of predicting environmental fluctuations.

The third assumption of the model is that agents are incapable of remembering or reusing behavioral innovations they adopted in the past. This assumption is implicit in the fact that the agents' directed rates of adaptation are the same in any direction, whether or not they previously explored that parameter range. This assumption is realistic for many animal species using only limited forms of social learning, so behavioral innovations gained by ancestors are forgotten as quickly as they become unfit. The assumption holds when there is no repertoire of past behavior from which the current generation can draw to deal with environmental conditions that occurred in the past.

We make the important caveat that we do not believe that the evolution of directed innovation, particularly in the vastly more complex system of humans, in fact occurred in the way we describe it here. Too many parameters, including the three assumptions stated above: the maximum rate of directed innovation, the existence of memory and forecasting abilities, remain unknown. We have, however, stated a theoretical possibility with clear and general implications. It can potentially provide insights into the cultural evolution of many animal species. Future work will provide more quantitative predictions that can be directly tested for particular species, cultural systems and environmental regimes.

## Supporting Information

Figure S1 **An example fitness curve for** *γ* = 4, *E* = 0, **and** *φ* **from** −5 **to** 5. It is very similar to the normal distribution curve but is easier to compute.
(TIFF)

Figure S2 **The environment used in the analytical model.** The environment increases in discrete steps of 1 each timestep for *c* timesteps, then drops to 0 in a single time step.
(TIFF)

Figure S3 **Agent behavior in this environment over the first cycle.** Dashed lines and diamonds indicate the phenotype of the agent, solid lines and circles the environment. The particlular *x* (maximum rate of phenotypic change) used here is *x* = 0.5, but changing *x* will only change the amplitude of the cycle, not its phase or frequency.
(TIFF)

Information S1. (PDF)
