# Supplementary figures and images for: Choosing Fitness-Enhancing Innovations Can Be Detrimental under Fluctuating Environments
Source: PLoS One. 2011 Nov 17;6(11):e26770. doi: 10.1371/journal.pone.0026770 (PMC3219637; doi:10.1371/journal.pone.0026770)

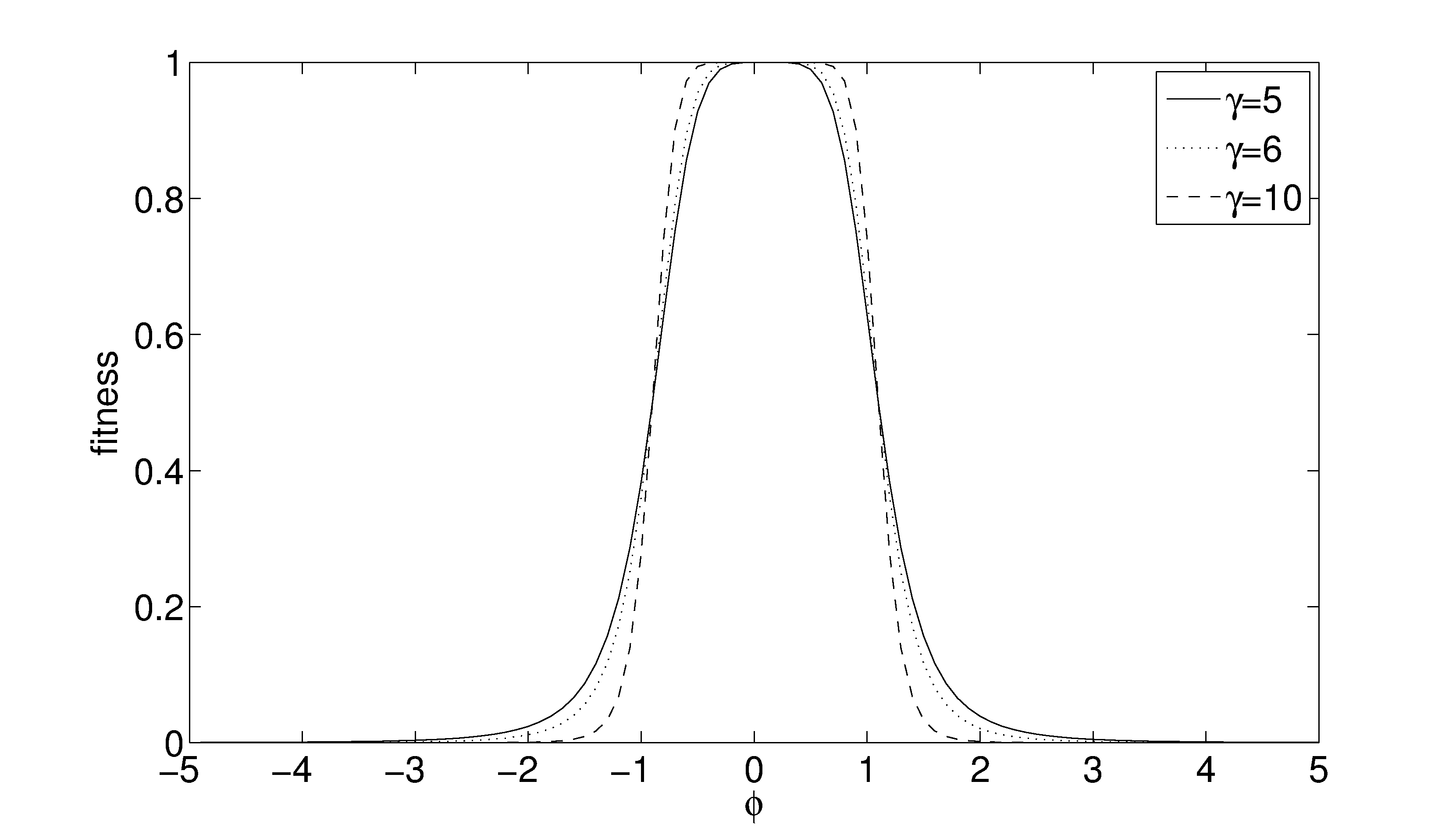

Supplement: Figure S1 — An example fitness curve for γ = 4, E = 0, and φ from −5 to 5. It is very similar to the normal distribution curve but is easier to compute. (TIFF) [file pone.0026770.s001.tiff]

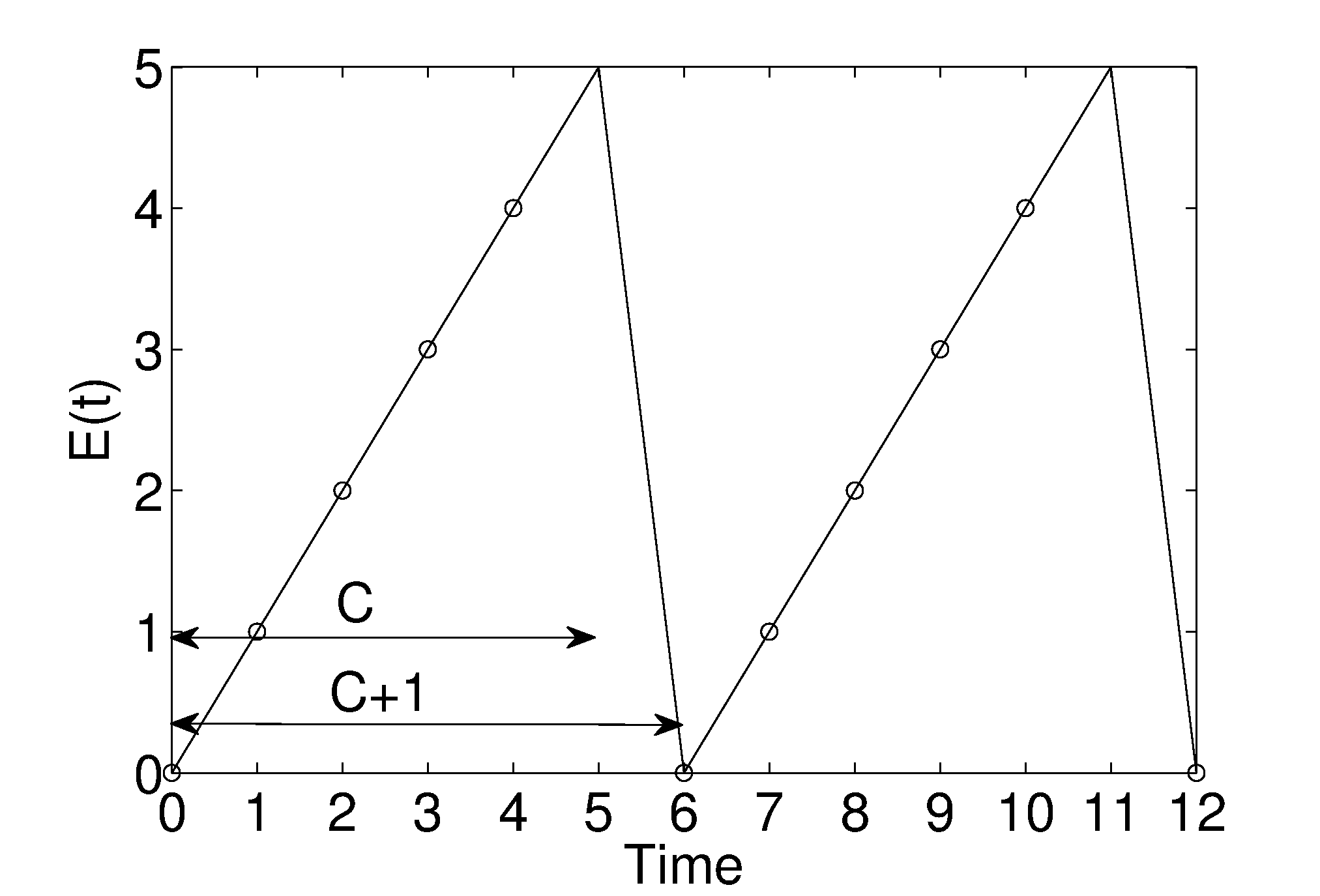

Supplement: Figure S2 — The environment used in the analytical model. The environment increases in discrete steps of 1 each timestep for c timesteps, then drops to 0 in a single time step. (TIFF) [file pone.0026770.s002.tiff]

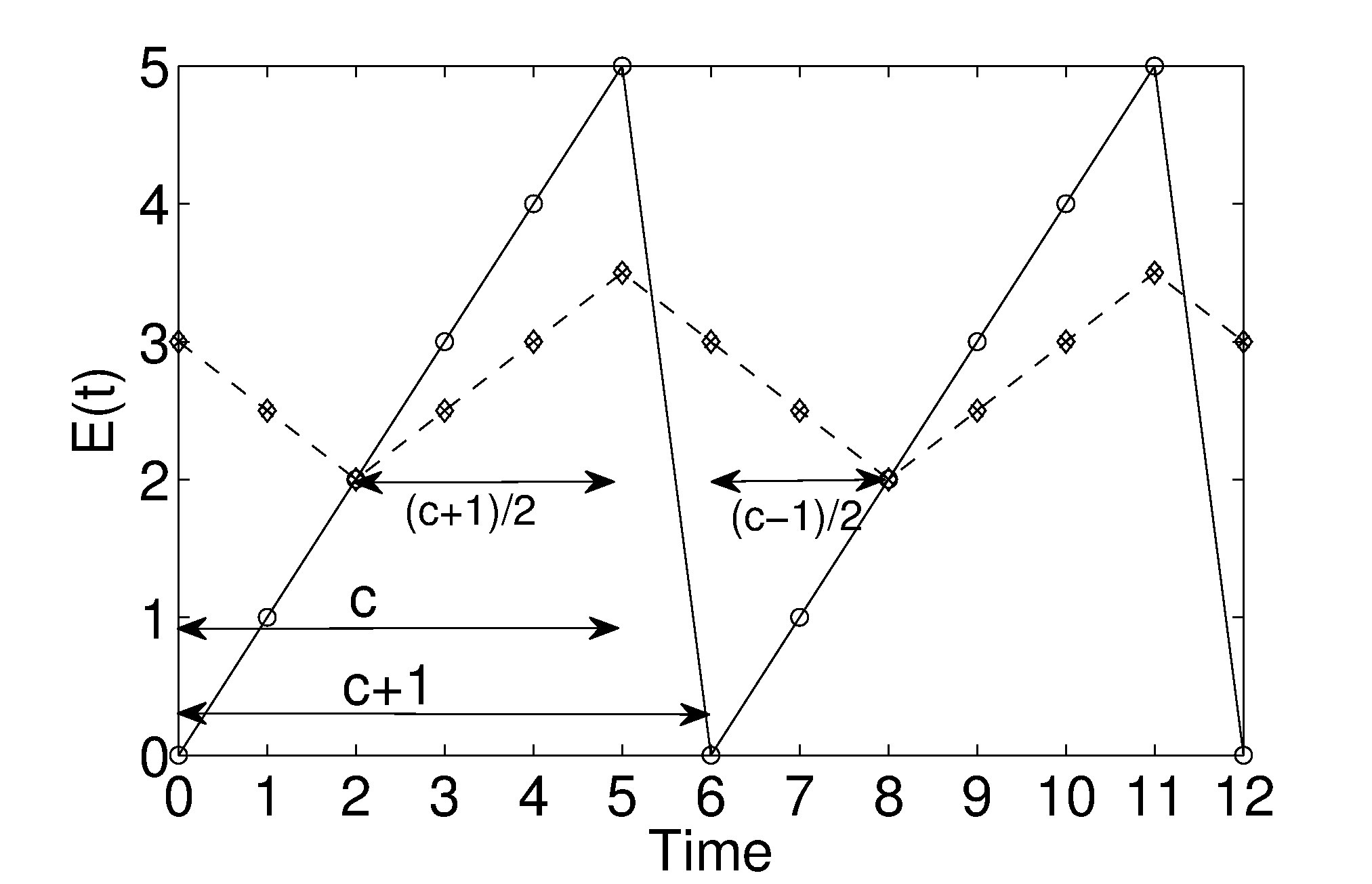

Supplement: Figure S3 — Agent behavior in this environment over the first cycle. Dashed lines and diamonds indicate the phenotype of the agent, solid lines and circles the environment. The particlular x (maximum rate of phenotypic change) used here is x = 0.5, but changing x will only change the amplitude of the cycle, not its phase or frequency. (TIFF) [file pone.0026770.s003.tiff]
