# Supplementary material for: Choosing Fitness-Enhancing Innovations Can Be Detrimental under Fluctuating Environments
Source: PLoS One. 2011 Nov 17;6(11):e26770. doi: 10.1371/journal.pone.0026770 (PMC3219637; doi:10.1371/journal.pone.0026770)
Supplement: Information S1. — (PDF) [file pone.0026770.s004.pdf]

Supporting information

Numerical example

By the fitness curve in equation (1), a perfectly fit population would have $\lambda = 1$, and if $|E(t) - \phi(t)| \to \infty$, then $\lambda \to 0$. Shape-wise, this fitness curve is very similar to the normal distribution curve that is ubiquitously used for this purpose, but has the advantage of being more tractable. An example fitness curve is shown in Figure F.1 for $\gamma = 4$, $E = 0$, and ϕ from -5 to 5.

The sequences of $\lambda_A(t)$ and $\lambda_B(t)$ are therefore:

$$\lambda_A(0) = 1, \qquad \lambda_A(1) = 1, \qquad \lambda_A(2) = 1, \qquad \lambda_A(3) = \frac{1}{2^\gamma + 1}, ...$$
$$\lambda_B(0) = 1, \qquad \lambda_B(1) = \frac{1}{0.5^\gamma + 1}, \qquad \lambda_B(2) = \frac{1}{1^\gamma + 1}, \qquad \lambda_B(3) = \frac{1}{1.5^\gamma + 1}, ...$$

The relative fitness of the two agents over time is simply the ratio of their geometric fitnesses:

$$\frac{\hat{\lambda}_A}{\hat{\lambda}_B}$$

which, due to the cyclicity of the environment, turns out to be simply:

$$\frac{\left(\frac{1}{2^\gamma + 1} \cdot\right)^{T/4}}{\left(\frac{1}{0.5^\gamma + 1} \cdot \frac{1}{1^\gamma + 1} \cdot \frac{1}{1.5^\gamma + 1}\right)^{T/4}}$$

where T is the total amount of time over which the two agents are compared. For ease of analysis, we assume that T is a multiple of 4. From this, it is easy to see that population B is more fit than population A if and only if

$$2^\gamma + 1 > 2 \cdot (0.5^\gamma + 1)(1.5^\gamma + 1)$$

A numerical solution to this is easily found: $\gamma > 3.237714...$

General case

To set up the problem, consider an environmental value $E(t)$ that begins at $E(0) = 0$ and increasing in increments of 1 in each discrete timestep for a total of $c > 1$ timesteps before it falls again to 0 at the timestep $t = c + 1$. The environment then repeats itself. The total cycle length is therefore $c + 1$ (see Figure F.2). Let x be the maximum amount that an agent can change to be closer to the current environmental optimum over one timestep. Clearly, if $x \geq c$, then the agent can adapt perfectly to this environment, since the environment changes at most a total of c units in a single timestep. Thus, we restrict $x < c$. In fact, for the following proof, we restrict $0 < x \leq 1$, so the most accurate possible agent can just track the environmental change during the slow upswing.

For the first problem, we wish to show that given two agents, A and B, under what conditions the geometric mean fitness of A is larger than the geometric mean fitness of B. By the same reasoning as in the numerical example, because the environment is cyclic, whether A or B is more fit depends on the ratio of their fitnesses over a single cycle of the environment. Since the behavior of both agents take on the same cycle-length as that of the environment, the future holds only repetitions of the same cycle and fitness differences are merely amplified. If we consider the first cycle, then the fitness of A in that period is:

$$\prod_{t=1}^{t<c+1} \frac{1}{a_t^\gamma + 1}$$

where $a_t = |E(t) - \phi_A(t)|$ is the misfit of the agent A at that time t. Similarly, the fitness of B is:

$$\prod_{t=1}^{t<c+1} \frac{1}{b_t^\gamma + 1}$$

where $b_t = |E(t) - \phi_B(t)|$. Over the first cycle, then, B is more fit than A if and only if:

$$\prod_{t=1}^{t<c+1} (b_t^\gamma + 1) < \prod_{t=1}^{t<c+1} (a_t^\gamma + 1)$$

We can expand the equation to get:

$$(b_1 b_2 ... b_c)^\gamma + ... + 1 < (a_1 a_2 ... a_c)^\gamma + ... + 1$$

The ellipsis between the + signs denote all possible combinations of products raised to the power γ. For

the lefthand side, they would be terms such as $(b_1b_2)^\gamma, (b_1b_3)^\gamma, (b_2b_3)^\gamma, (b_1b_2b_3)^\gamma$, etc. If we let $\mathbf{T}$ be the set of time steps over the first cycle, so $\mathbf{T} = \{1, 2, ..., c+1\}$ and if we denote $\mathcal{P}(\mathbf{T})$ be the the power set over $\mathbf{T}$ (that is, the set of all possible subsets of timesteps in the first cycle), and we let $\mathbf{t}$ be one subset of $\mathbf{T}$, then the above can be written:

$$\sum_{\mathbf{t}\in\mathcal{P}(\mathbf{T})} \left(\prod_{t\in\mathbf{t}} b_t\right)^\gamma + 1 < \sum_{\mathbf{t}\in\mathcal{P}(\mathbf{T})} \left(\prod_{t\in\mathbf{t}} a_t\right)^\gamma + 1 \tag{A.1}$$

The 1's cancel, and a sufficient, but not necessary, condition for B to be more fit than A, when γ is large enough, is if the maximum among the set $\prod_{t\in\mathbf{t}} b_t$ is smaller than the maximum among the set $\prod_{t\in\mathbf{t}} a_t$. One possible (although, again not necessary) way this can happen is if this is the product of all the misfits when the phenotypic distance to the environmental optimum is greater than 1. Thus, if we define $\mathbf{t_A}$ be the maximal set of time steps such that $a_t > 1 \forall t \in \mathbf{t_A}$, and similarly define $\mathbf{t_B}$, then equation (A.1) is true if (but not only if) the following condition holds:

$$\prod_{t\in\mathbf{t_B}} b_t < \prod_{t\in\mathbf{t_A}} a_t$$

and γ is large enough.

This is because for γ large enough, this maximal term will dominate all other terms in equation (A.1).

The arbitrary threshold of 1 in the condition $a_t > 1 \forall t \in \mathbf{t_A}$ and $b_t > 1 \forall t \in \mathbf{t_B}$ can be relaxed by introducing another parameter, δ, into the fitness equation, such that it looks like:

$$\lambda = \frac{1}{|\delta(E(t) - \phi(t))|^\gamma + 1} \tag{A.2}$$

In this way, the threshold that must be achieved to be considered as among the "worst" period is simply $\frac{1}{\delta}$.

What this result means is that to understand which agent among A and B are superior, we need to compare the product of the greatest (and not necessarily consecutive) misfits for both agents. If this product is greater for A than for agent B, then agent B will be more fit than agent A. If we know that $x_A > x_B$, that is, A has more foresight than B, then for the environment in Figure F.2, we know that at the very least the single epsiode of greatest misfit for agent A will will be greater than the comparable one for agent B (just after the reversal). Thus, B will be more fit than A for a range of δ, and γ large

enough. For the agent-based simulations, we use $\delta = 1$, although the qualitative results are quite robust to δ, the reason of which can be intuited from the analysis in the following section.

The existence of a local optimum

We have so far shown that if $x_A > x_B$, then there is some range of γ and δ for which B is more fit than A. Now we will show that for any γ and environmental variable c, there exists at least one local optimum x_{crit} which is optimal between $x = 0$ and $x = 1$. We will do this for $\delta = 1$, but the proof follows for any δ. The curious fact is that x_{crit} exists and is not maximal.

Consider how an agent with $x \in [0, 1]$ changes its phenotype $\phi(t)$ in the same environment as considered previously. Since the environment moves in cycles and agent behavior is entirely forced by the environment, it is reasonable to expect that the agent will also settle down in cycles. Since the agent cannot fully adapt to the environment in either the upswing or the downswing, we expect it to accelerate at its maximum directedness, x, both when the environment is above it as well as when the environment is below it. This amounts to finding a cyclical behavior of cycle length $c + 1$ where the agent increases its phenotypic value at rate x for half the cycle, and decreases its phenotypic value at rate x for half the cycle. To make our life simpler, we assume c is odd, so $c+1$ is even. The agent settles in the cycle shown in Figure F.3.

Again by the same reasoning as used in the numerical example, to find the x that maximizes the geometric mean fitness over time means finding the x that maximizes the geometric mean fitness over a single cycle. If we phase shift the environment so that at time $t = 0$, $E(0) = \phi(0)$, we see in the Figure F.3 the first cycle.

It is not too difficult to see that on the upswing of both the environmental value and the agent phenotype, the difference between the agent phenotype and the environmental value at any point t is $t(1-x)$. After the environment swings down, the agent phenotype also swings down until the environment catches up again, a process that takes exactly as much time as the upswing, since the agent is moving at the same rate in either direction. The calculation for the downswing is a little more involved, but it is not difficult to see that the maximum value that $\phi(t)$, the agent phenotype, attains is:

$$\frac{c-1}{2} + \frac{c+1}{2}x$$

The $(c-1)/2$ portion can be seen from Figure F.3, since this is the point at which $\phi(t)$ and $E(t)$ equilibriates. The $(c-1)/2 \cdot x$ portion is simply because $\phi(t)$ moves upward at rate x for $(c+1)/2$ timesteps.

From this maximum value, $\phi(t)$ descends at rate of x per timestep, while the environment rises from 0 at the rate of 1 per timestep. Hence the different between the environment and the phenotype at this phase is simply:

$$\frac{c-1}{2} + \frac{c+1}{2}x - tx - (t-1)$$

Which nicely reduces down to

$$(1+x)\left(\frac{c+1}{2} - t\right)$$

If we use the same fitness curve as previously (see Equation (1)), the product of the fitnesses over an entire cycle turns out to be:

$$U(x) = \prod_{t=1}^{\frac{c+1}{2}} \frac{1}{[t(1-x)]^\gamma + 1} \cdot \frac{1}{\left[(1+x)\left(\frac{c+1}{2} - t\right)\right]^\gamma + 1} \tag{A.3}$$

We let U represent the product of all fitnesses over one cycle. We begin at $t = 1$ since for the upswing, $t = 0$ makes no difference, while the first step of the downswing is the same step as the last step of the upswing, and so the first timestep for that period is also discounted.

We wish to show that there is an intermediate level of x which maximizes equation (A.3). This can be done in the usual way by taking its derivative, but it is obviously much more easily done in terms of the log of the function. Since (A.3) is strictly positive, we can take its logarithm, and since log is an strictly increasing function, maximizing (A.3) is equivalent to maximizing its log,

$$\log U(x) = -1 \cdot \sum_{t=1}^{\frac{c+1}{2}} \left(\log[t^\gamma (1-x)^\gamma + 1] + \log\left[(1+x)^\gamma \left(\frac{c+1}{2} - t\right)^\gamma + 1\right]\right) \tag{A.4}$$

Taking the derivative of this results in

$$\frac{d \log U(x)}{dx} = \sum_{t=1}^{\frac{c+1}{2}} \left(\frac{\gamma t^\gamma (1-x)^{\gamma-1}}{t^\gamma (1-x)^\gamma + 1} - \frac{\gamma (1+x)^{\gamma-1} \left(\frac{c+1}{2} - t\right)^\gamma}{(1+x)^\gamma \left(\frac{c+1}{2} - t\right)^\gamma + 1}\right) \tag{A.5}$$

We now look for the 0's of this equation and showing there is a maximum to equation (A.4). If $x = 1$, it's quite easy to see that the first term of the summation disappears and the function is negative. If $x = 0$, it is a little harder to see that the function is positive. If we let $x = 0$, then equation (A.5) reduces down to:

$$\left.\frac{d\log U(x)}{dx}\right|_{x=0} = \sum_{t=1}^{\frac{c+1}{2}}\left(\frac{\gamma t^\gamma}{t^\gamma+1}\right) - \sum_{t=1}^{\frac{c+1}{2}}\left(\frac{\gamma\left(\frac{c+1}{2}-t\right)^\gamma}{\left(\frac{c+1}{2}-t\right)^\gamma+1}\right) \tag{A.6}$$

If we write out the second term:

$$\frac{\gamma\left(\frac{c+1}{2}-1\right)^\gamma}{\left(\frac{c+1}{2}-1\right)^\gamma+1} + \frac{\gamma\left(\frac{c+1}{2}-2\right)^\gamma}{\left(\frac{c+1}{2}-2\right)^\gamma+1} + ... + \frac{\gamma\left(\frac{c+1}{2}-\frac{c+1}{2}\right)^\gamma}{\left(\frac{c+1}{2}-\frac{c+1}{2}\right)^\gamma+1}$$

We can reverse the order and rewrite it as

$$\frac{\gamma\cdot 0^\gamma}{0^\gamma+1} + \frac{\gamma\cdot 1^\gamma}{1^\gamma+1} + ... + \frac{\gamma\left(\frac{c-1}{2}\right)^\gamma}{\left(\frac{c-1}{2}\right)^\gamma+1}$$

Which is equivalent to

$$\sum_{t=0}^{\frac{c-1}{2}}\left(\frac{\gamma t^\gamma}{t^\gamma+1}\right)$$

Thus, equation (A.6) reduces to

$$\begin{aligned}\left.\frac{d\log U(x)}{dx}\right|_{x=0} &= \sum_{t=1}^{\frac{c+1}{2}}\left(\frac{\gamma t^\gamma}{t^\gamma+1}\right) - \sum_{t=0}^{\frac{c-1}{2}}\left(\frac{\gamma t^\gamma}{t^\gamma+1}\right)\\ &= \frac{\gamma\left(\frac{c+1}{2}\right)^\gamma}{\left(\frac{c+1}{2}\right)^\gamma+1} > 0\end{aligned}$$

We have so far shown that equation (A.5) is positive if $x = 0$ and is negative if $x = 1$. We also note that $\log U(x)$ is continuous and differentiable over this range. Thus, $\log U(x)$ is guaranteed a maximum $x_{crit} \in [0, 1]$. However, since its derivative is negative at $x = 1$, there exists an $\epsilon > 0$ such that $\log U(1-\epsilon) > \log U(1)$, so $x = 1$ cannot be a maximum. Similarly, $x = 0$ cannot be a maximum. Thus,

there is a maximum x_{crit} strictly between $]0,1[$, where $][$ denotes that the boundaries are not included in the set. Since $\log U$ is an increasing function of U, this x_{crit} is a local maximum for $U(x)$ also. In this instance, then, it does not always pay, fitness wise, to be more directed by increasing x, since there is at least one local optimum in x. Note that this result made no assumptions on γ, and that the same reasoning carries through if we used a different δ with the fitness curve (A.2).

Figure Legends

Figure F.1. An example fitness curve for $\gamma = 4$, $E = 0$, and ϕ from -5 to 5. It is very similar to the normal distribution curve but is easier to compute.

Figure F.2. The environment used in the analytical model. The environment increases in discrete steps of 1 each timestep for c timesteps, then drops to 0 in a single time step.

Figure F.3. Agent behavior in this environment over the first cycle. Dashed lines and diamonds indicate the phenotype of the agent, solid lines and circles the environment. The particlular x (maximum rate of phenotypic change) used here is $x = 0.5$, but changing x will only change the amplitude of the cycle, not its phase or frequency.
